# Rubisco and Rubisco Activase Play an Important Role in the Biochemical Limitations of Photosynthesis in Rice, Wheat, and Maize under High Temperature and Water Deficit

**DOI:** 10.3389/fpls.2017.00490

**Published:** 2017-04-13

**Authors:** Juan A. Perdomo, Sebastià Capó-Bauçà, Elizabete Carmo-Silva, Jeroni Galmés

**Affiliations:** ^1^Plant Biology and Crop Science, Rothamsted Research (BBSRC)Harpenden, UK; ^2^Research Group on Plant Biology under Mediterranean Conditions, Universitat de les Illes Balears-INAGEAPalma de Mallorca, Spain; ^3^Lancaster Environment Centre, Lancaster UniversityLancaster, UK

**Keywords:** crops, photosynthesis, Rubisco, Rubisco activase, temperature, water deficit

## Abstract

To understand the effect of heat and drought on three major cereal crops, the physiological and biochemical (i.e., metabolic) factors affecting photosynthesis were examined in rice, wheat, and maize plants grown under long-term water deficit (WD), high temperature (HT) and the combination of both stresses (HT-WD). Diffusional limitations to photosynthesis prevailed under WD for the C_3_ species, rice and wheat. Conversely, biochemical limitations prevailed under WD for the C_4_ species, maize, under HT for all three species, and under HT-WD in rice and maize. These biochemical limitations to photosynthesis were associated with Rubisco activity that was highly impaired at HT and under HT-WD in the three species. Decreases in Rubisco activation were unrelated to the amount of Rubisco and Rubisco activase (Rca), but were probably caused by inhibition of Rca activity, as suggested by the mutual decrease and positive correlation between Rubisco activation state and the rate of electron transport. Decreased Rubisco activation at HT was associated with biochemical limitation of net CO_2_ assimilation rate (*A*_N_). Overall, the results highlight the importance of Rubisco as a target for improving the photosynthetic performance of these C_3_ (wheat and rice) and C_4_ (maize) cereal crops under increasingly variable and warmer climates.

## Introduction

As a consequence of climate change, global temperatures have increased over the last few decades and this warming trend is predicted to accelerate in the near future (IPCC, 2013). Increases in global temperatures are often accompanied by alterations in precipitation patterns, with effects on the amount, intensity, frequency and type of precipitation (Dore, 2005). The changing global climate is expected to have a detrimental effect on agriculture by increasing the prevalence of abiotic stresses.

Heat and drought are the principal abiotic stresses limiting plant growth and crop productivity. Photosynthesis, the main physiological process driving plant growth, is highly sensitive to drought and heat stress (Chaves et al., 2009; Mathur et al., 2014; Singh et al., 2014), especially when both stresses are imposed together (Carmo-Silva et al., 2012; Vile et al., 2012; Perdomo et al., 2015). Photosynthetic CO_2_ assimilation can be constrained by diffusive and biochemical limitations (Flexas and Medrano, 2002a; Pinheiro and Chaves, 2011). The diffusive limitations are a consequence of stomatal closure (i.e., decreased stomatal conductance, *g*_s_) and increased leaf resistance to CO_2_ transport from the atmosphere to the site of carboxylation (i.e., decreased mesophyll conductance, *g*_m_), as generally observed under mild to moderate water deficit (WD) (Chaves et al., 2003, 2009; Flexas et al., 2004; von Caemmerer and Evans, 2010).

The biochemical or metabolic components that limit photosynthesis under WD are less well described than the diffusion limitations (Galmés et al., 2007b). Metabolic limitations to photosynthesis under drought have been associated with impaired ATP synthesis (Tezara et al., 1999; Flexas et al., 2004; Singh et al., 2014), which is due to a decrease in the electron transport rate (J) (Flexas et al., 1999; Galmés et al., 2007a). Lower ATP availability, in turn, affects ribulose-1,5-bisphosphate (RuBP) regeneration, thus limiting the rate of CO_2_ fixation. The effects of drought stress on Rubisco vary depending on the plant species and intensity of stress; some studies reported a dramatic reduction in Rubisco activity (Parry et al., 2002; Zhou et al., 2007) while others showed little or no inhibition of the enzyme (Panković et al., 1999; Pelloux et al., 2001). A meta-analyses suggested that Rubisco did not limit photosynthesis until severe or long-term drought stress was encountered (Flexas et al., 2006a). More recently, Galmés et al. (2011) suggested that low chloroplastic CO_2_ concentration (*C*_c_) occurring under WD could induce de-activation of Rubisco in some Mediterranean species.

High leaf temperatures affect both electron transport capacity (*J*_max_) and the maximum rate of carboxylation of Rubisco (*V*_cmax_) (Dreyer et al., 2001; Yamori et al., 2006, 2008). On the contrary, data in literature suggest that high temperatures (HTs) do not sufficiently impair *g*_s_ and *g*_m_ to cause diffusion components to significantly limit photosynthesis (Bernacchi et al., 2002; Evans and von Caemmerer, 2013; Walker et al., 2013; von Caemmerer and Evans, 2015). Moderately HTs impair the activation of Rubisco by its catalytic chaperone, Rubisco activase (Rca), which becomes the primary cause of the decrease in photosynthesis in response to elevated temperature (Crafts-Brandner and Salvucci, 2000; Salvucci and Crafts-Brandner, 2004; Kim and Portis, 2005; Galmés et al., 2013). In addition to Rubisco activation, moderately HTs can also inhibit electron transport activity, ATP synthesis, and RuBP regeneration (Schrader et al., 2004; Yamori et al., 2008; Carmo-Silva and Salvucci, 2011). As the temperature increases further above the thermal optimum and reaches non-physiological conditions, photosynthesis may be increasingly limited due to impairment of the physical integrity of electron transport components of the photosynthetic apparatus (Salvucci and Crafts-Brandner, 2004).

The above described effects of HT on the photosynthetic processes are mainly based on studies where measurements were done at HT in plants grown at a moderate (control) temperature. Although there is abundant evidence that photosynthesis can acclimate to temperature (Gunderson et al., 2000; Way and Yamori, 2014; Yamori et al., 2014), little is known about the effects of high growth temperature on the relative contribution of diffusive and biochemical limitations to photosynthesis. If biochemical limitations prevailing at HTs of measurement also predominate at HTs of growth, the analysis of Rubisco and Rca performance and thermal acclimation may provide valuable information toward the improvement of crop photosynthesis at HTs.

The activity of Rubisco is regulated by Rca, which facilitates the dissociation of inhibitory sugar phosphates from the active site of Rubisco in an ATP-dependent manner (Spreitzer and Salvucci, 2002). Most species studied to date, including rice and wheat, contain two isoforms of Rca, a shorter redox-insensitive β-isoform of 41–43 kDa and a longer redox-sensitive α-isoform of 46–48 kDa (Zhang and Portis, 1999). Some species, such as maize and tobacco, however, do not appear to contain significant amounts of the longer redox-sensitive α-isoform (e.g., Salvucci et al., 1987). Changes in the redox status and ADP/ATP ratio of the chloroplast modulate the activity of Rca, thereby mediating the regulation of Rubisco activation and net CO_2_ assimilation in response to the prevailing irradiance (Salvucci et al., 1985; Mott and Woodrow, 2000; Carmo-Silva and Salvucci, 2013; Scales et al., 2014). The activity of Rca is extremely thermally sensitive. This enzyme becomes inactive, decreasing the rate of net CO_2_ assimilation at moderately HTs.

The objective of the present study was to test the hypothesis that decreased Rubisco activation state limits photosynthesis under heat stress, and heat stress combined with WD, in the C_3_ cereals rice and wheat and the C_4_ cereal maize. The effects of long-term plant growth under WD, HT and the combination of both (HT-WD) were therefore investigated on Rubisco activity and amount, Rubisco activase content and Rubisco activation state and to relate them with the relative contributions of biochemical and diffusive limitations to photosynthesis in rice, wheat and maize.

## Materials and Methods

### Plant Material, Growth Conditions, and Treatments

Rice (*Oryza sativa* L. cv. Bomba), wheat (*Triticum aestivum* L. cv. Cajeme) and maize (*Zea mays* L. cv. Carella) plants were grown from seeds in a greenhouse in 3.5 L pots containing a 70:30 mixture (v:v) of horticultural substrate (60% Fine blonde peat, 40% Fine black peat, granulometry 0–10 mm, 3.5 kg/m^3^ calcium dolomite and 1.12% of N – 0.2% of P_2_O_5_ – 0.2% of K_2_O plus 1.45% of microelements; Prohumin 6040, Projar S.A, Spain) and perlite (granulometry A13, Projar S.A, Spain). After 2 weeks, the seedlings were selected to uniform size with 1 plant per pot in maize, and 10 plants per pot in wheat and rice. Thereafter, the plants were moved to a controlled environment room. Light was provided by metal halide lamps (OSRAM, Germany) placed at specific distances from the plants to obtain a photosynthetically active photon flux density (PPFD) of 500 μmol m^-2^ s^-1^, with a photoperiod of 12 h day/12 h night. The ambient temperature and the relative humidity were monitored with portable sensors Testo 175-H1 data logger (Gerilab, Spain). The relative humidity was maintained between 40 and 60% using humidifiers.

For logistical reasons, the plants were grown in two sets, which were subjected to each of the two temperature treatments. A first set of plants of the three species was grown at the control temperature (Control, 25/20°C; VPD, 1.8/1.0 kPa day/night); and a second set of plants was grown at HT (38/33°C; VPD, 3.5/2.3 kPa day/night). Only temperature and VPD differed between the two sets of plants or experiments, while all other environmental conditions (e.g., light intensity and quality, air removal, photoperiod duration) were identical and computer-controlled.

For each set of plants, i.e., for each growing temperature and VPD treatment, ten pots per species were grown at soil field capacity until plants had developed fully expanded leaves (typically 2 weeks). Thereafter, 20 days after germination, pots of all species were randomly assigned to two different irrigation treatments: five pots per species were maintained at 100% field capacity during the whole experiment (well-watered treatment, WW) and the other five pots were maintained at 45% field capacity (moderate WD treatment, WD), as determined by pot weighing every day and compensating the daily water losses with 50% Hoagland’s solution that provided all necessary nutrients for the plant. The soil water availability for plants under WD was determined with respect to the control by measuring the water weight in five representative samples of the substrate mixture used in the experiment. Plants were considered to be under WD when *g*_s_ was decreased by 40% compared to the well-watered plants; *g*_s_ was considered as a good indicator of the WD status, as previously demonstrated (Medrano et al., 2002). Therefore, a total of four treatments were established: 25°C of growth temperature and well-watered (control), 25°C of growth temperature and WD, 38°C of growth temperature and well-watered (HT) and 38°C of growth temperature and water-deficit (HT-WD).

New leaves were allowed to develop and expand under the two irrigation treatments for a minimum of 30 days. All measurements and samples were taken 40–50 days after the water treatment was initiated (i.e., 60–70 days after germination), on new leaves developed completely under the temperature and/or water treatments (Perdomo, 2015). Plants of all three species were in the vegetative stage and analyses used young fully expanded leaves.

Leaf samples for biochemical measurements were collected at mid-morning (4 h after the beginning of the photoperiod). Leaf disks of 0.5 cm^2^ were quickly frozen into liquid nitrogen and stored at -80°C until extraction. These samples were used for the following determinations: Rubisco initial and total activity, activation state and amount, and Rubisco activase amount.

### Gas Exchange and Chlorophyll a Fluorescence Measurements

All leaf gas exchange and chlorophyll *a* fluorescence measurements were performed on the youngest fully expanded leaf of each plant, using a portable photosynthesis system (Li-6400-40; Li-Cor Inc., USA) equipped with a leaf chamber fluorometer (Li-6400-40, Li-Cor Inc.), the latter using the multi-flash protocol (Loriaux et al., 2013). The net CO_2_ assimilation rate (*A*_N_) and the stomatal conductance (*g*_s_) were measured at mid-morning at a leaf temperature of 25°C, saturating PPFD of 1500 μmol m^-2^ s^-1^ (provided by the light source of the Li-6400-40, with 10% blue light), a CO_2_ concentration in the leaf chamber (*C*_a_) of 400 μmol CO_2_ mol^-1^ air and a relative humidity between 40 and 50%. A PPFD of 1500 μmol m^-2^ s^-1^ was considered to provide photosynthesis saturation for the glasshouse grown plants (Makino et al., 1994; Grassi and Magnani, 2005; Centritto et al., 2009; Ghannoum, 2009; Tazoe et al., 2009; Zhu et al., 2012; Xiong et al., 2015). The leaf dark respiration rate (*R*_dark_) was determined at pre-dawn (i.e., shortly before the start of the light period) at a *C*_a_ of 400 μmol CO_2_ mol^-1^ air. The gross CO_2_ assimilation rate (*A*_G_) was calculated from the sum of *A*_N_ and half of *R*_dark_ (Bermúdez et al., 2012).

The photochemical efficiency of photosystem II (ΦPSII) was determined according to Genty et al. (1989):

$$\Phi \text{PSII=}({F^{\prime }}_{m}-F_{s})/{F^{\prime }}_{m}$$

where *F*_s_ is the steady-state fluorescence yield and *F*_m_′ the maximum fluorescence yield obtained with a light-saturating pulse of 8000 μmol m^-2^ s^-1^.

The linear rate of electron transport (J) was calculated according to Krall and Edwards (1992):

$$J=\Phi_{\text{PSII}}\cdot \text{PPFD}\cdot \alpha \cdot \beta$$

where α is the leaf absorbance and β is the partitioning of absorbed quanta between photosystems I and II. β was assumed to be 0.5 for the C_3_ species (Laisk and Loreto, 1996; Tosens et al., 2012) and 0.4 for maize (von Caemmerer, 2000). α was measured for all species grown under each treatment inside a dark chamber using the light source from the Li-6400-40 and a spectroradiometer (HR2000CG-UV-NIR; Ocean Optics Inc., USA), as described by Schultz (1996). All values obtained for α were 0.86–0.87, with non-significant differences between species and species × treatment combinations.

### Estimation of *C*_c_, *C*_s_, and *g*_m_

From combined gas-exchange and chlorophyll *a* fluorescence measurements, the mesophyll conductance to CO_2_ (*g*_m_) was estimated for wheat and rice using the so-called variable J method (Harley et al., 1992). The estimated value of *g*_m_ for wheat and rice, both C_3_ species, was used to calculate *C*_c_ by applying the equation:

$$C_{c}=C_{i}-\left(A_{N}/g_{m}\right)$$

Maize has a C_4_-based carbon concentrating mechanism, with inherent complexity that complicates mathematical modeling (Collatz et al., 1992; von Caemmerer and Furbank, 1999; von Caemmerer, 2000; Ubierna et al., 2012). In this study, both *g*_m_ and *g*_bs_ (bundle sheath conductance) were considered constant in maize (von Caemmerer, 2000; Massad et al., 2007; Ghannoum, 2009). Yin et al. (2016) have recently shown large variation in *g*_bs_ in response to measurement temperature in maize plants grown at a constant temperature of 27°C. To the best of our knowledge, there are no reports on the variation of *g*_bs_ with growth temperature. Furthermore, a sensitivity analysis (results not shown) demonstrated that even large changes in *g*_m_ did not affect our results; we expect the same would hold true for *g*_bs_. The CO_2_ concentration in the bundle sheath (*C*_s_) of maize leaves was estimated from the hyperbolic function describing the *A*_N_-*C*_i_ curves using the C_4_ photosynthesis model described by von Caemmerer (2000) as detailed by Massad et al. (2007) and with the modifications of Perdomo et al. (2016).

### Quantification of Photosynthetic Limitations

To compare the relative limitations to CO_2_ assimilation induced by WD, HT and the combination of both stresses, the photosynthetic limitations were partitioned into their functional components following the approach proposed by Grassi and Magnani (2005). This approach uses values for *A*_G_, *g*_s_, and *g*_m_ (Supplementary Table S1) and the maximum rate of Rubisco carboxylation (*V*_cmax_) as references. The maximum *A*_G_, concomitantly with *g*_s_ and *V*_cmax_, was reached under control conditions, therefore the control treatment was used as a reference. In the present study, *V*_cmax_ was calculated as the product of the Rubisco amount, the activation state and the carboxylase catalytic turnover rate (*k*_cat_^c^) measured *in vitro* at 25°C (2.1, 2.2 and 4.1 s^-1^ for rice, wheat, and maize, respectively; Perdomo et al., 2016). Thereafter, the photosynthetic limitations were partitioned into components related to diffusion, i.e., stomatal (*S*_L_) and mesophyll limitations (MC_L_), and leaf Rubisco-based biochemistry (*B*_L_), estimated using the next equations:

$$D_{L}=S_{L}+{MC}_{L}$$

$$B_{L}∼V_{\text{cmax}}$$

The analysis of biochemical limitations in maize was restricted to the C_3_ cycle activity. Data obtained under control conditions was used as the reference.

### Rubisco Activity and Amount in Leaf Crude Extracts

Rubisco was extracted by grinding three leaf disk samples (total area of 1.5 cm^2^) in a mortar with 500 μL of ice-cold extraction buffer containing 50 mM Bicine-NaOH pH 8.0, 1 mM ethylene diamine tetracetic acid (EDTA), 5% (w/v) polyvinylpyrrolidone (PVP), 6% polyethylene glycol (PEG_4000_)_,_ 50 mM β-mercaptoethanol, 10 mM dithiothreitol (DTT) and 1% (v/v) protease-inhibitor cocktail (Sigma–Aldrich Co. LLC., USA). Leaf extracts were then centrifuged at 14000 × *g* for 1 min at 4°C. The supernatant was kept at 4°C and used immediately for the measurement of Rubisco activity and amount.

The activities of Rubisco were determined by the incorporation of ^14^CO_2_ into acid-stable products at a reaction temperature of 25°C for plants grown both at control and HT, following the protocol described in Parry et al. (1997). The reaction mixture (500 μL) contained 100 mM Bicine-NaOH pH 8.2, 20 mM MgCl_2_, 10 mM NaH^14^CO_3_ (15.54 kBq μmol^-1^) and 0.1 mM RuBP. The initial activity was determined by adding 10 μL of crude extract to the reaction mixture. The total activity was measured after incubating 10 μL of the same extract for 3 min with all the components except RuBP, to allow carbamylation of all available Rubisco catalytic sites, and then starting the reaction by adding RuBP. All reactions were quenched after 60 s by adding 100 μL of 10 M HCOOH. The activation state of Rubisco was obtained as the ratio between the initial and total activities. All quenched reaction mixtures were completely dried at 100°C, the residues dissolved in 400 μL H_2_O, mixed with 3.6 mL of Ultima Gold scintillation cocktail (PerkinElmer Inc., USA) and radioactivity due to the ^14^C stable products determined in a liquid scintillation counter (LS-6500, Beckman Coulter Inc., USA).

The amount of Rubisco was measured by electrophoresis (Aranjuelo et al., 2005). One aliquot of the leaf crude extract was mixed with loading buffer, consisting of 65 mM Tris-HCl pH 6.8, 3 M sucrose, 0.6 M β-mercaptoethanol, 5% (w/v) sodium dodecyl sulphate (SDS), and 0.01% bromophenol blue. Samples were heated at 96°C for 5 min and then allowed to cool at room temperature. The total soluble protein (TSP) concentration in the crude extracts was determined by the method of Bradford (1976). A volume representing 15 μg of TSP per sample (crude extract mixed with loading buffer) was loaded onto a 12.5% SDS-polyacrylamide gel (12.5% resolving, 4% stacking; 0.75 mm thick; Bio-Rad Laboratories Inc., USA). This amount of protein was within the range of linear response of optical density for known concentrations of Rubisco purified from wheat (standard used for calibration). The solubilized proteins were separated by SDS–PAGE (Laemmli, 1970) with electrophoresis being carried out at room temperature at a constant voltage (200 V). The gels were fixed in 500:150:75 (v/v/v) water–methanol–acetic acid mixture for 1 h, stained in EZ Blue Gel Staining (Sigma–Aldrich Co. LLC., USA) solution for 1 h and subsequently rinsed in water to remove excess stain. Finally, the gels were scanned with a high-resolution scanner (HP Scanjet G3010, Hewlett Packard, Spain) and the amount of large Rubisco subunit was determined by densitometry with the image analysis software TotalLab v2005 (Non-linear Dynamics, USA).

### Rubisco Activase Protein Amount

The relative amount of Rca was measured by immunoblotting after separation of proteins by SDS–PAGE (Supplementary Figure S1; Salvucci et al., 2001). Soluble proteins were extracted from samples consisting of three leaf disks (total area of 1.5 cm^2^) by grinding in a mortar with 500 μL of ice-cold extraction buffer containing 50 mM Tricine-NaOH pH 8.0, 10 mM EDTA, 1% (w/v) PVP, 20 mM β-mercaptoethanol, 1 mM phenylmethylsulfonyl fluoride (PMSF), 10 μM leupeptin and 1% (v/v) protease-inhibitor cocktail. The leaf extracts were centrifuged at 14000 × *g* for 1 min at 4°C and 25 μL of the supernatant was rapidly added to 20 μL loading buffer (described above). After determination of the TSP concentration in the crude extracts, sample aliquots of extracts plus loading buffer corresponding to 6 μg of TSP were loaded onto a 12.5% SDS-polyacrylamide gel (Bio-Rad Laboratories Inc., USA) and separated by electrophoresis at 100 V. Serial dilutions of extracts prepared from leaf disks taken from plants of each species under control conditions were used as standards, by loading 5, 10, and 15 μg of TSP. SDS-PAGE gels were blotted onto nitrocellulose membranes in 50 mM Trizma base/50 mM boric acid for 1 h at 100 V within the Mini-Protean system (Bio-Rad Laboratories Inc., USA). Following blocking with 4% (w/v) non-fat milk, blots were probed with monospecific antibodies (Salvucci et al., 2001). Immunodetection of Rca protein via colorimetry was carried out with the BCIP/NBT alkaline phosphatase system according to the manufacturer’s instructions (Sigma–Aldrich Co. LLC., USA). The relative amount of Rubisco activase in each sample was determined by whole-band analysis of the membrane using an image acquisition densitometer (ChemiDoc XRS+ system, Bio-Rad Laboratories Inc., USA), with the image analysis software Quantity One v4.6.5 (Bio-Rad Laboratories Inc., USA).

### Statistical Analysis

The statistical significance of trait variation was tested by factorial ANOVA, with species, irrigation treatments and growth temperatures as fixed factors, and the interaction between treatments. *Post hoc* comparison between treatments was performed using the Duncan test (*P* < 0.05) in the Statistica 6.0 software package (StatStof Inc., USA). Regression coefficients were calculated with the 11.0 Sigma Plot software package (Systat Software Inc., Germany).

## Results

### Photosynthetic Limitations in Cereals under Water Deficit and High Temperature

The effects of WD and high growth temperature (HT) on the growth and physiology of rice, wheat and maize were addressed in previous studies (Perdomo et al., 2015, 2016). The detrimental effects of these two stresses on the gross CO_2_ assimilation rate (*A*_G_) and stomatal (*g*_s_) and mesophyll conductance (*g*_m_) are shown in Supplementary Table S1. These data were used, together with maximum Rubisco carboxylation activity, to determine the contribution of the different types of limitations to photosynthesis under WD, HT, and HT-WD combination.

**FIGURE 1:**
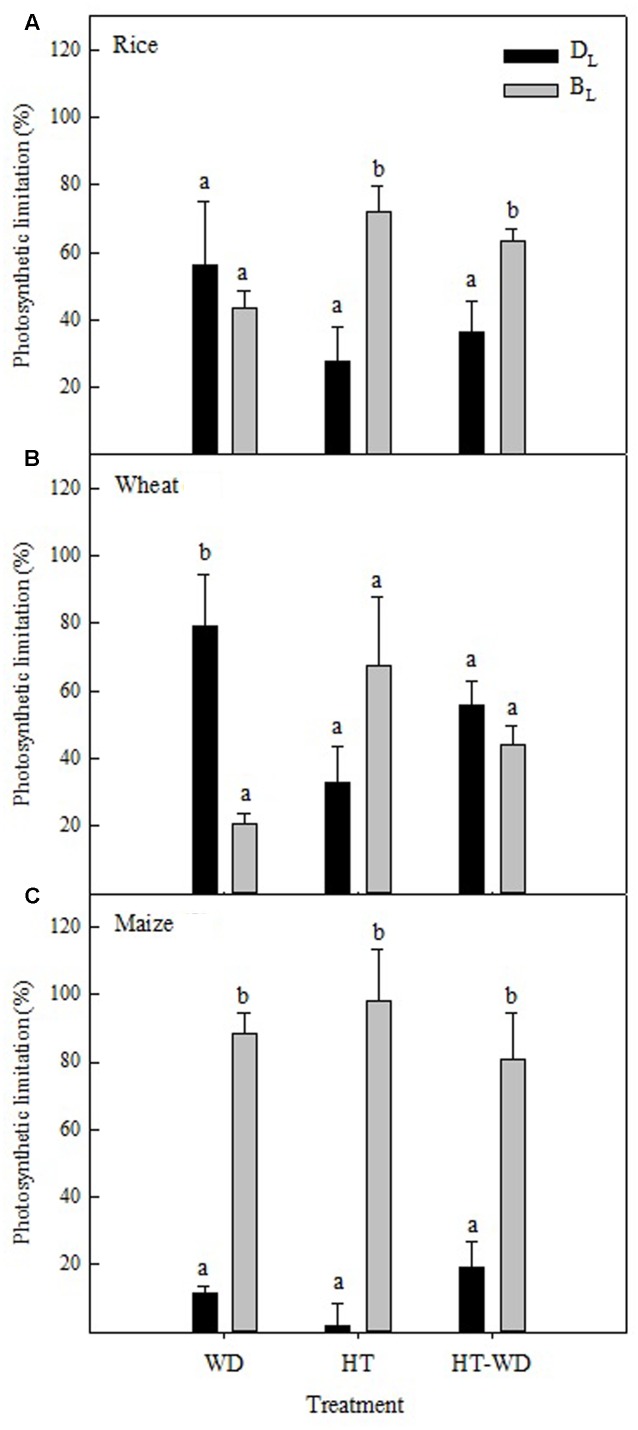
**The diffusive (*D*_L_) and biochemical limitations (*B*_L_) to CO_2_ assimilation in rice** **(A)**, wheat **(B)**, and maize **(C)** plants grown under water deficit (WD), high temperature (HT) and a combination of HT and water deficit (HT-WD). Values represent means ± SE (*n* = 4–5). Different letters denote statistically significant differences by Duncan analysis (*P* < 0.05) between types of limitation within each species and treatment.

Under WD, the diffusive limitations (*D*_L_) accounted for most of the photosynthetic limitations in wheat, while the biochemical limitations (*B*_L_) were predominant in maize and both types of limitations had a similar contribution in rice (**Figure 1A**). Importantly, the analysis of the biochemical limitations in maize was restricted to the C_3_ cycle activity, taking into account those limitations associated with Rubisco, and not with the C_4_ cycle activity, including phosphoenolpyruvate carboxylase (PEPC). Under HT and the combination of the two stresses (HT-WD), the contribution of *B*_L_ was larger than that of *D*_L_ and explained most of the inhibition of the photosynthetic CO_2_ assimilation in rice and maize, whereas both *B*_L_ and *D*_L_ limitations contributed equally to the inhibition of photosynthesis in wheat under HT-WD (**Figures 1B,C**).

**FIGURE 2:**
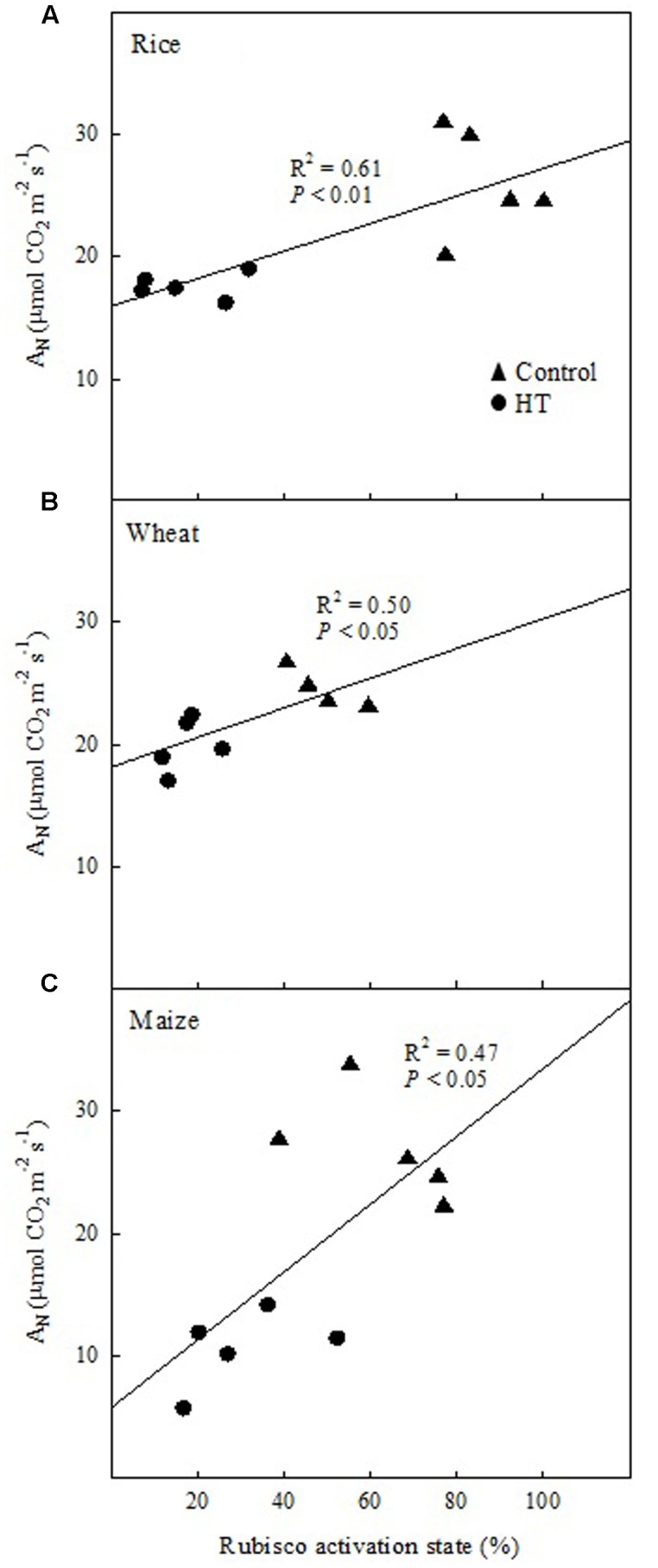
**The relationship between the Rubisco activation state and the net CO_2_ assimilation rate (*A*_N_) in well-watered plants of rice** **(A)**, wheat **(B)**, and maize **(C)** grown at 25°C (control) or 38°C (HT) and measured at 25°C. Each symbol corresponds to one independent sample.

The relationship between the net CO_2_ assimilation rate (*A*_N_) and the *in vitro* Rubisco activation provided further evidence for the observed photosynthetic limitations. At HT, the prevalence of *B*_L_ in the three species was confirmed by the positive correlation of *A*_N_ vs. Rubisco activation state in well-watered plants grown at 25°C or 38°C and measured at 25°C (**Figure 2**). Maize and rice showed decreases in *A*_N_ and Rubisco activation state with the increase in temperature (**Figure 2**). Under WD and HT-WD, the relationship, *A*_N_ vs. Rubisco activation state, was positive in rice (*R*^2^ = 0.51, *P* < 0.05, data not shown), but not in wheat and maize (*P* > 0.05, data not shown), in agreement with the limitation analysis (**Figure 1A**).

### Rubisco Amount and Activities in Cereals under Water Deficit and High Temperature

**FIGURE 3:**
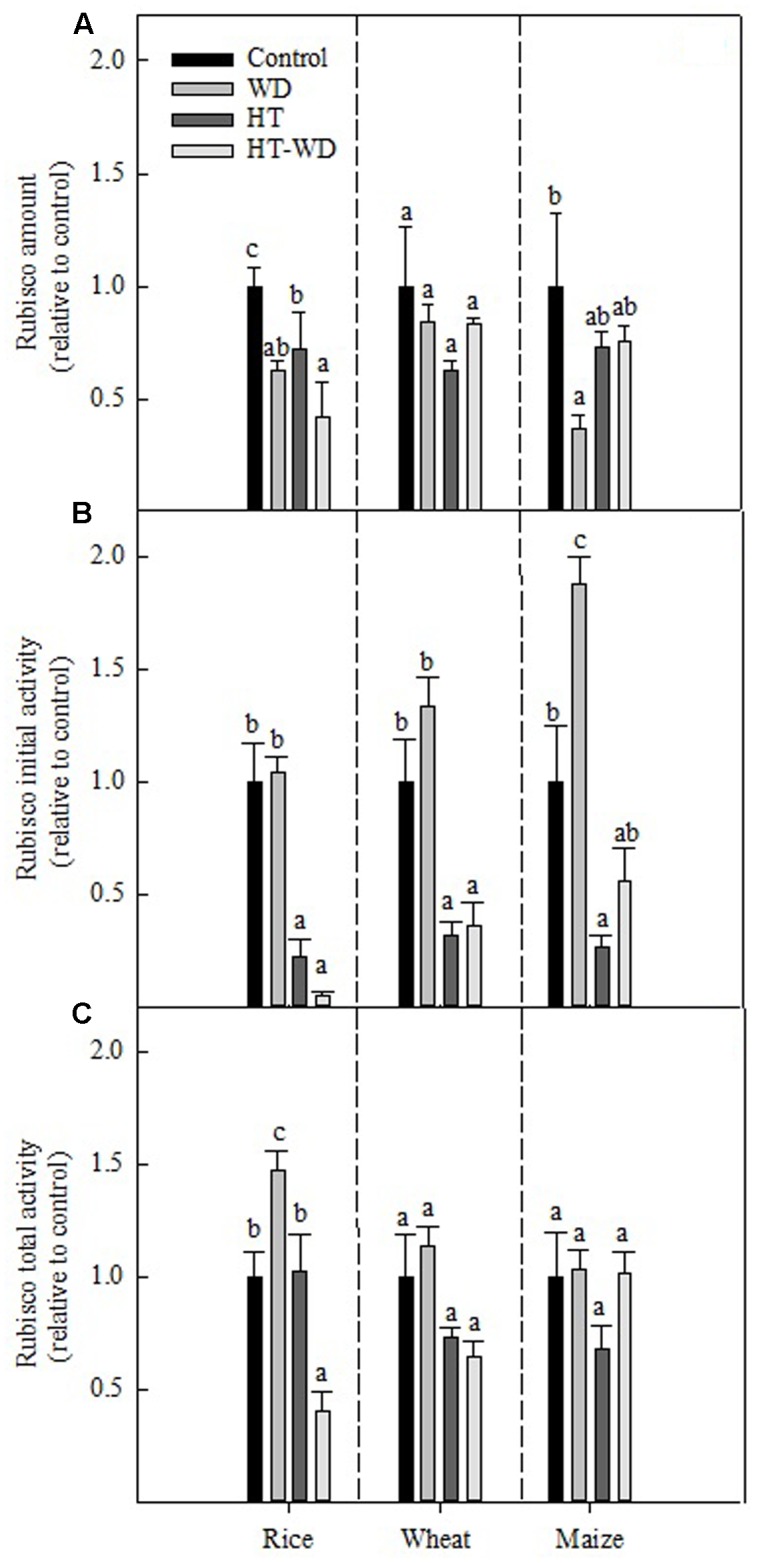
**Rubisco amount** **(A)**, initial **(B)** and total **(C)** activities at 25°C measured in plants of rice, wheat, and maize grown at control, WD, HT and a combination of HT and water deficit (HT-WD) conditions. To unify scales among the different species, values are means ± SE (*n* = 4–5) of each parameter expressed relative to control plants. Different letters denote statistically significant differences by Duncan analysis (*P* < 0.05) among treatments within each species. The control values for rice, wheat, and maize of Rubisco amount were, respectively, 0.49 ± 0.03, 0.34 ± 0.04, 0.15 ± 0.05 mg Rubisco mg^-1^ TSP; Rubisco initial activity 0.31 ± 0.02, 0.19 ± 0.04, 0.08 ± 0.01 μmol CO_2_ mg^-1^ TSP min^-1^; and Rubisco total activity 0.36 ± 0.01, 0.44 ± 0.05, 0.13 ± 0.02 μmol CO_2_ mg^-1^ TSP min^-1^.

Water deficit and HT stresses affected the amount and activities of Rubisco in rice, wheat and maize differently, depending on the treatment and the species (**Figure 3**). Results are relative to the values obtained for control plants to facilitate comparison among the three species. While the amount of Rubisco in wheat was not affected by any of the applied treatments, it decreased in rice and maize under WD and in rice plants grown at HT (**Figure 3A**). The combined HT-WD treatment was no more detrimental than the each of the individual stresses for any of the species; rice was the species with the largest decrease in Rubisco amount, with ca. 50% less Rubisco under HT-WD compared to the control.

Rubisco initial activity was not affected negatively by WD in any of the three species (**Figure 3B**). In fact, maize showed an increase in the initial activity, to almost the double under WD compared to the control treatment. By contrast, Rubisco initial activity decreased severely in plants of the three species grown under HT. The combination HT-WD was not more detrimental than HT on its own, which suggests that Rubisco initial activity is more sensitive to inhibition by HT than by WD in these three species. As observed with the amount of Rubisco, rice showed the largest decrease in the initial activity of Rubisco under the combined stress treatment.

Rubisco total activity was less affected than the initial activity under the applied treatments (**Figure 3C**). In rice, Rubisco total activity decreased only under HT-WD and non-significant effects were observed in wheat and maize. Overall, the different response between the initial and total activities indicates that the applied treatments affected the Rubisco activation state, particularly under HT and HT-WD (**Figures 3B,C**).

### Rubisco Activase Amount in Cereals under Water Deficit and High Temperature

**FIGURE 4:**
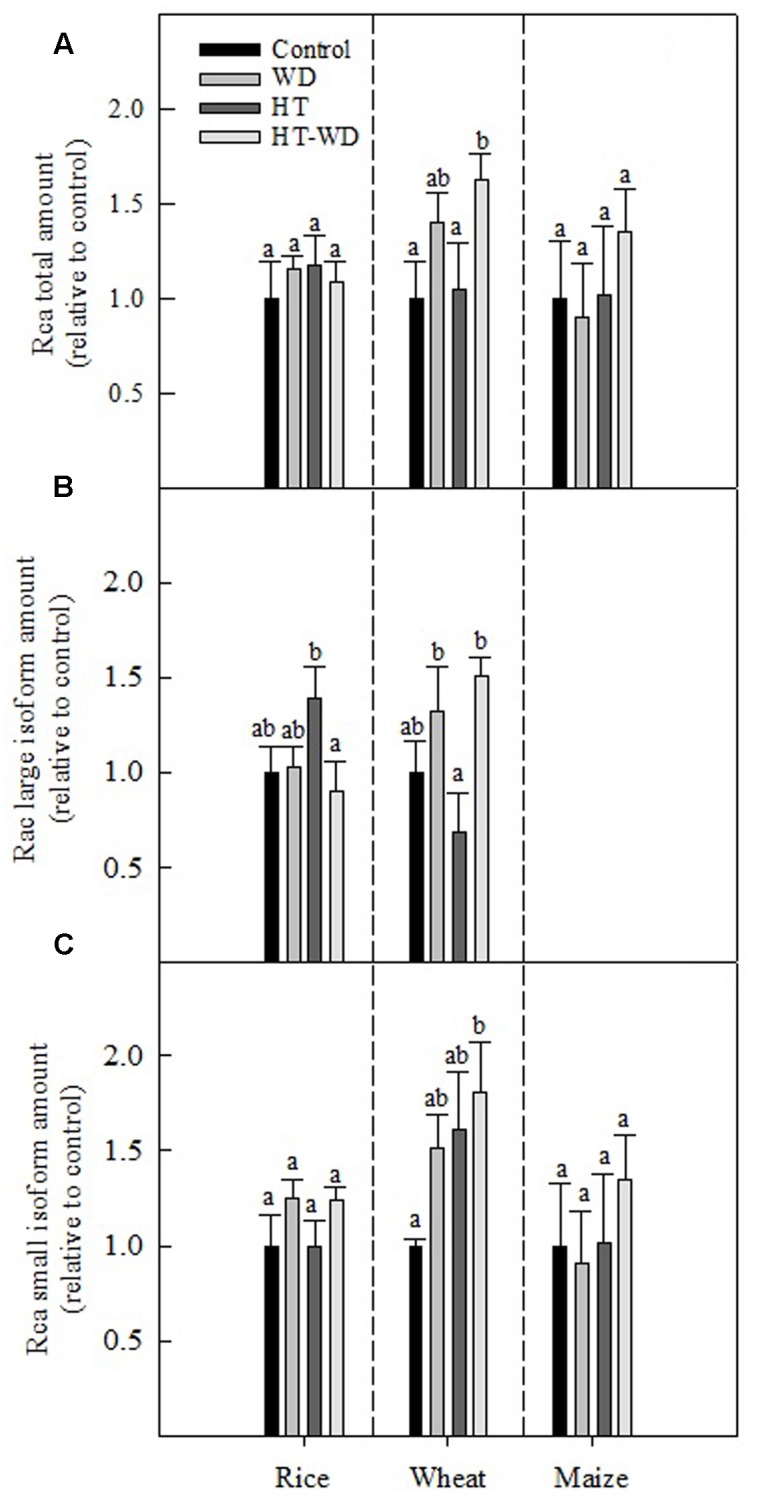
**Total Rubisco activase (Rca) amount** **(A)**, Rca large isoform amount **(B)** and Rca small isoform amount **(C)** in plants of rice, wheat, and maize grown at control, WD, HT and a combination of HT and water deficit (HT-WD) conditions. Values represent means ± SE (*n* = 4) of amounts expressed relative to control plants. Different letters denote statistically significant differences by Duncan analysis (*P* < 0.05) among treatments within each species.

The total amount of Rca relative to plants grown under control conditions was not significantly affected by WD and HTs, except in wheat where Rca increased in plants exposed to the combination HT-WD treatment (**Figure 4A**). With the exception of wheat, the Rca amount was constant under the different treatments, which indicates that the decrease in Rubisco activity was not due to a decrease in the total Rca amount. However, when the large and small Rca isoforms were quantified separately, some differences among treatments and species became apparent. The Rca large isoform was observed only in the two C_3_ species; in rice the amount was higher at HT than HT-WD, whereas in wheat the amount was higher under WD and HT-WD than under HT alone (**Figure 4B**). The results suggest that the Rca large isoform is susceptible to HT in wheat. The amount of the small Rca isoform did not show significant differences among the treatments in rice and maize. Conversely, in wheat the amount of the small isoform increased considerably under the combined stresses HT-WD compared to control plants (**Figure 4C**).

### Rubisco Activation Dependence on the CO_2_ Availability, Rubisco and Rca Amounts, and Rate of Electron Transport

**FIGURE 5:**
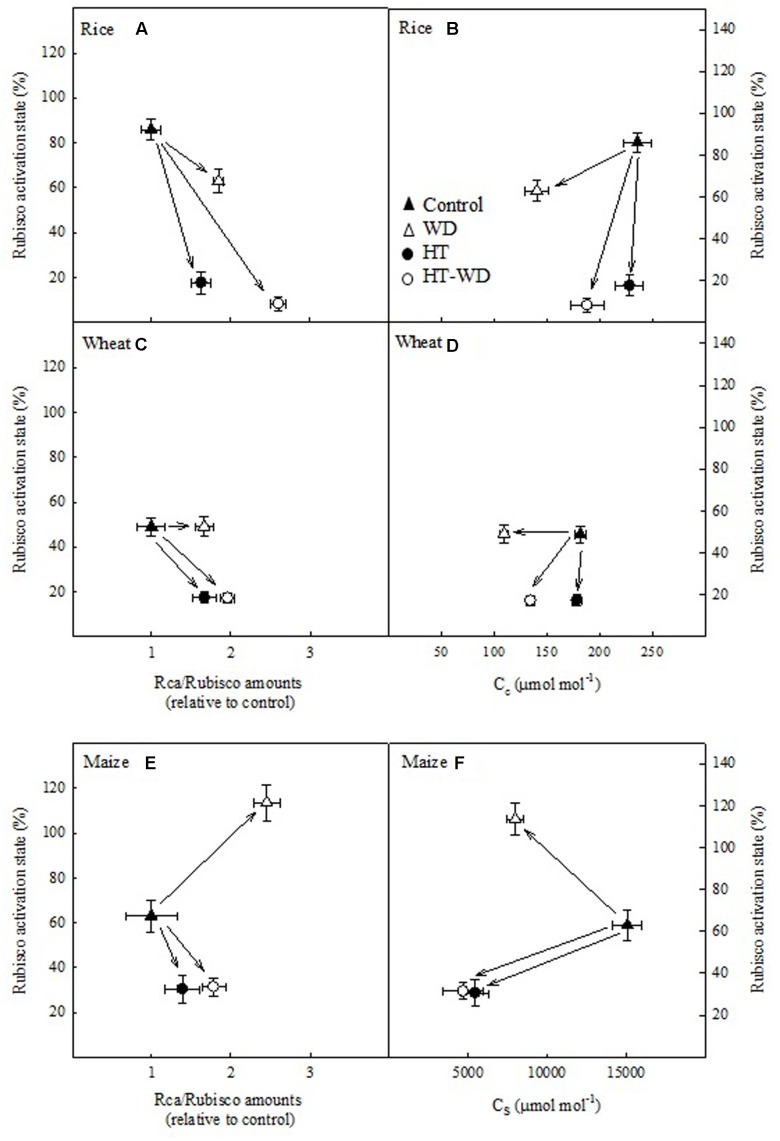
**Rubisco activation state in relation to the ratio of Rubisco activase (Rca) to Rubisco amounts (Rca/Rubisco;** **A,C,E)**, the CO_2_ concentration in the mesophyll chloroplasts (*C*_c_; **B,D)** or the bundle sheath (*C*_s_; **F)** in rice **(A,B)**, wheat **(C,D),** and maize **(E,F)**. Values represent means ± SE (*n* = 4–5).

The activation state of Rubisco was plotted in relation to the ratio of Rca/Rubisco amounts and to the concentration of CO_2_ in the chloroplast of the mesophyll and the bundle sheath cells (*C*_c_ and *C*_s_) in the two C_3_ species and maize, respectively (**Figure 5**). Wheat and rice exhibited a similar pattern; under WD the decrease in the activation state of Rubisco was minor (in rice) or non-existent (in wheat), and were accompanied by moderate increases in the ratio of Rca/Rubisco amounts and decreases in *C*_c_ (**Figures 5A–D**). Rice and wheat plants grown under HT stress showed large decreases in Rubisco activation state, alongside with modest increases in the Rca/Rubisco amounts and no changes in *C*_c_. Maize presented a similar pattern to that observed in the C_3_ species, with the exception of WD plants which exhibited an increase in the activation state of Rubisco and a large increase in the ratio of Rca/Rubisco amounts (**Figure 5E**).

**FIGURE 6:**
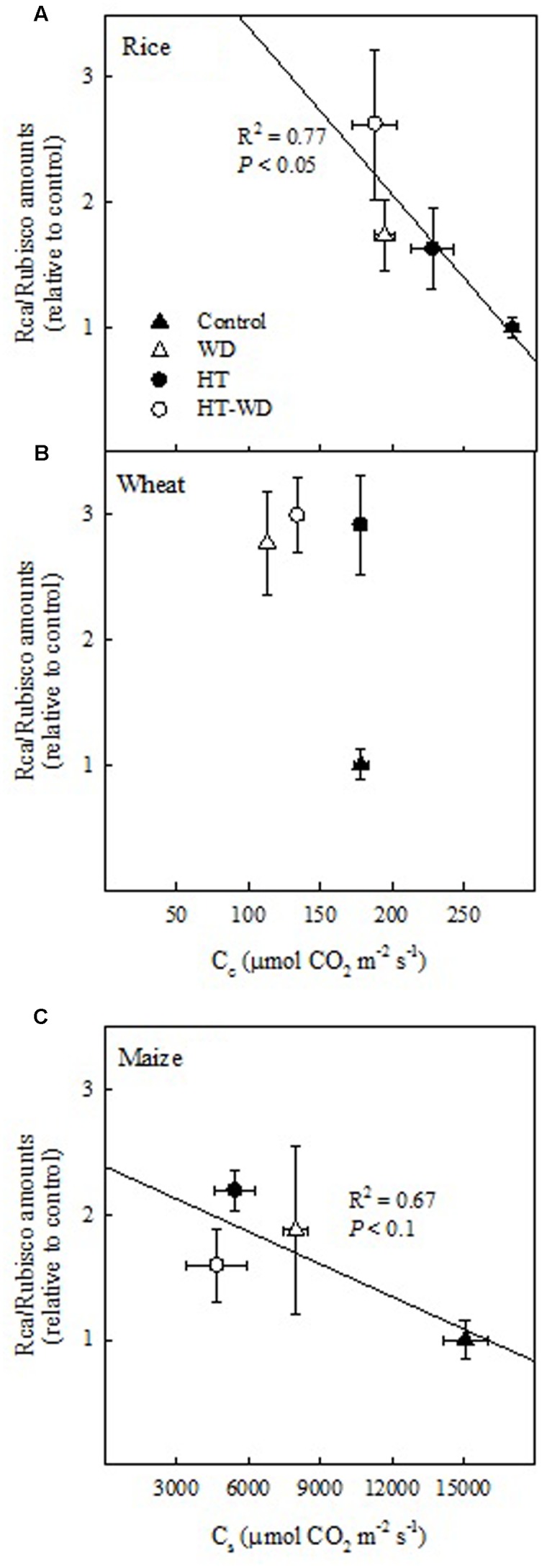
**The relationship between the CO_2_ concentration in the mesophyll chloroplasts (*C*_c_) in rice** **(A)** and wheat **(B)** and the CO_2_ concentration in the bundle sheath chloroplasts (*C*_s_) in maize **(C)** and the ratio of Rubisco activase (Rca) to Rubisco amounts (Rca/Rubisco). Values represent means ± SE (*n* = 4–5).

A positive relationship between the activation state of Rubisco and the ratio of Rca/Rubisco amounts would be expectable as there is more Rca to activate Rubisco. However, the results above suggest that changes in the activation of Rubisco are due to the combined effects of adjustments in the ratio of Rca/Rubisco amounts and in *C*_c_ or *C*_s_. In fact, increases in the ratio of Rca/Rubisco amounts correlated with decreases in *C*_c_ in rice (*P* < 0.05) and with decreases in *C*_s_ in maize (*P* < 0.1) (**Figure 6**). This correlation, which was not observed in wheat, suggests that rice and maize adjusted the ratio of Rca/Rubisco amounts to the concentration of CO_2_ available for carboxylation, however, wheat varies Rca but not Rubisco amounts under the different treatments (**Figure 3**).

**FIGURE 7:**
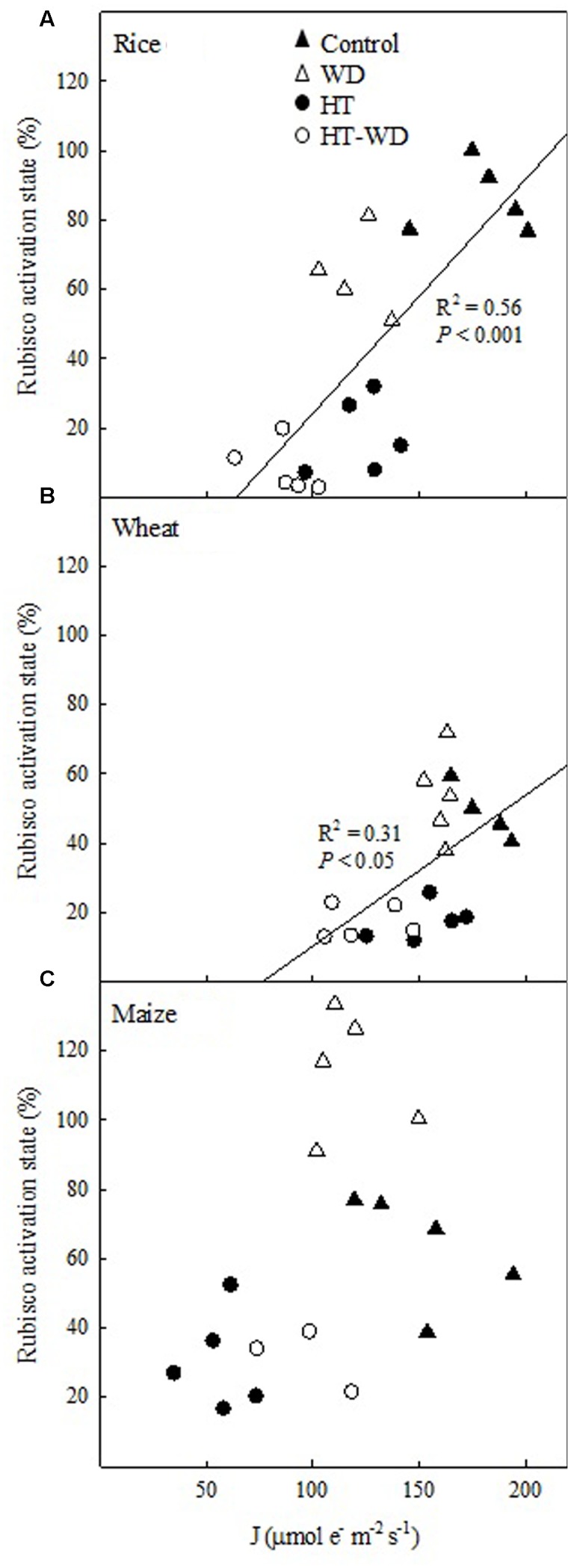
**The relationship between the Rubisco activation state and the electron transport rate (J) in rice** **(A)**, wheat **(B)**, and maize **(C)**.

Rubisco activation state showed a significant positive correlation with the electron transport rate (J) in the two C_3_ species (**Figures 7A,B**). In rice and wheat, J and Rubisco activation state decreased when the growth temperature increased, independently of the irrigation treatment. However, rice showed a slight decrease in J and Rubisco activation state under WD at both growth temperatures, while wheat did not show any differences between well-watered and WD within each growth temperature. Therefore, rice was the species most affected by the combined HT-WD treatment. Although maize did not show a significant correlation between Rubisco activation state and J, the same pattern was apparent, with a decrease in both parameters at HT independent of the watering treatment (**Figure 7C**).

## Discussion

Water deficit and heat stress are two main factors adversely affecting crop productivity. The effects of these stresses, independently and in combination, on the physiological responses of three main cereals, wheat, rice and maize were examined in previous studies (Perdomo et al., 2015, 2016). In the present manuscript, the focus was on the response of the CO_2_-fixing enzyme, Rubisco, and of its molecular chaperone Rca. Additionally, physiological and biochemical data were combined to assess the type of limitations to photosynthesis under these two stresses. Although there was more than one plant per pot in rice and wheat, all plants had plentiful supply of nutrients for growth so as to avoid any interference with the effect of the stresses here studied.

### Photosynthesis Is Impaired by Diffusion Limitations under Water Deficit and Biochemical Limitations under High Temperature in Rice, Wheat, and Maize Plants Subjected to Long-term Stressful Conditions

The results showed that diffusional limitations (*D*_L_) constrained CO_2_ assimilation, at least in the two C_3_ species under WD, whereas biochemical limitations (*B*_L_) were associated with the inhibition of photosynthesis under heat stress in all three species (**Figure 1**). These findings are in agreement with previous reports for other species (Chaves et al., 2003; Pinheiro and Chaves, 2011; Carmo-Silva et al., 2012).

Under WD, both of the C_3_ species exhibited reduced stomatal conductance (*g*_s_), while a decrease in mesophyll conductance (*g*_m_) was also observed in rice (Supplementary Table S1). Hence, decreased capacity to transfer CO_2_ from the atmosphere to the chloroplast stroma under WD imposed a limitation on photosynthesis in the C_3_ species (**Figure 1**). Decreased *g*_s_ and *g*_m_ under WD have been shown to limit the CO_2_ concentration at the Rubisco site in the mesophyll cells (*C*_c_) of C_3_ species and in the bundle-sheet cells (*C*_s_) in C_4_ species (Flexas and Medrano, 2002a; Chaves et al., 2003; Ghannoum, 2009; Lopes et al., 2011). This finding was confirmed in the present study (**Figure 5**). In rice, the lower concentration of CO_2_ imposed a biochemical limitation by decreasing the activation state of Rubisco (**Figure 5**), which explains the similar contribution of *D*_L_ and *B*_L_ under WD (**Figure 1**). On the contrary, decreased *C*_c_ in wheat under WD did not result in lower Rubisco activation state, which may explain why *B*_L_ were less prominent in this species (**Figure 1**). These results suggest that Rubisco in rice is more sensitive to de-activation than wheat Rubisco at low CO_2_ availability. Different sensitivities of Rubisco de-activation under limiting C_c_ have been reported among species from contrasting environments (Galmés et al., 2011). In both C_3_ species, rice and wheat, the ratio ETR/*A*_G_ increased under WD (data not shown). This behavior is agreement with reports from literature in a large number of species (Flexas and Medrano, 2002b; Medrano et al., 2002; Salazar-Parra et al., 2012) indicating an increase in photorespiration under WD conditions.

Growth at HT did not alter *C*_c_, but decreased the activation state of Rubisco in rice and wheat (**Figure 5**), in agreement with the predominant role of *B*_L_ under HT (**Figure 1**). A recent report indicated that leaf conductances tend to remain unchanged and/or increase at measuring temperatures up to 40°C in rice and wheat plants grown at optimum temperatures (von Caemmerer and Evans, 2015). In our study, no changes were observed in *g*_s_ in rice and wheat plants grown at HT and measured at 25°C, and *g*_m_ decreased only in rice (Supplementary Table S1).

The analysis of limitations of the C_3_ cycle – Rubisco dependent – in maize revealed that *B*_L_ prevailed both under WD and HT (**Figure 1**), suggesting that the observed decrease in *C*_s_ under WD was not limiting to CO_2_ assimilation rates (Supplementary Table S1 and **Figure 5**). Rubisco in maize was markedly affected by WD (decreased amount) and HT (decreased Rubisco activation state) (**Figure 3**). This decrease in Rubisco activation state in HT-grown maize was related to marked inhibition of photosynthetic capacity (**Figure 2**), as previously reported in this species (Crafts-Brandner and Salvucci, 2002; Sharwood et al., 2016). Although the analysis of photosynthetic limitations did not take into account the enzymes of the C_4_ cycle in maize, two of the key enzymes of C_4_ photosynthesis, pyruvate phosphate dikinase (PPDK) and PEPC, are insensitive to leaf temperatures up to 40°C in maize (Crafts-Brandner and Salvucci, 2002). Therefore, the results reported in the present study are in agreement with the notion that Rubisco regulation makes C_4_ photosynthesis as sensitive to inhibition by heat stress as C_3_ photosynthesis (Crafts-Brandner and Salvucci, 2002; Ghannoum, 2009; von Caemmerer and Furbank, 2016), despite the fact that the C_4_ CO_2_-concentrating mechanism offers a greater buffering capacity against HT and the diffusion limitations under water stress.

Rubisco initial activity was also markedly affected in plants of all three species under the combined effect of HT-WD (**Figure 3**), which has been previously observed in different cotton cultivars (Carmo-Silva et al., 2012). In rice and maize, *B*_L_ were predominant under the combined treatment whereas in wheat, both *D*_L_ and *B*_L_ contributed to inhibit photosynthesis when the two stresses were imposed together (**Figure 1**). It is known that WD and HT limit photosynthesis in C_3_ (Flexas et al., 2004; Hu et al., 2010) and C_4_ species (Ripley et al., 2007; Ghannoum, 2009). While little is known about the detrimental effect of the combination of these two stresses, in the few studies where these effects have been measured, photosynthesis was highly sensitive to the combination of HT-WD (Prasad et al., 2008, 2011; Silva et al., 2010; Vile et al., 2012).

### Biochemical Limitations Are Mainly Attributed to Changes in the Rubisco Activation State via Adjustments in the Concentration of CO_2_, Rubisco/Rca Relative Amounts and Rca Activity

To understand the effects of WD and HTs on photosynthesis, it is important to elucidate the biochemical components that are affected, particularly those associated with the Rubisco enzyme. WD effects on Rubisco are still unresolved, with some studies showing no effect (Vapaavuori, 1986; Pelloux et al., 2001) and others reporting decreases in Rubisco content and activation (Flexas et al., 2006b; Galmés et al., 2011). Some reports show that decreases in the Rubisco content and activity are associated with the severity of WD and are species-specific (Parry et al., 2002; Tezara et al., 2002; Bota et al., 2004). In rice and maize, but not in wheat, the amount of Rubisco decreased under WD, but Rubisco initial and total activities increased in maize and rice, respectively (**Figure 3**). Other authors have reported a decrease in the initial and total activities of Rubisco that has been attributed to a decrease in the Rubisco content (Flexas and Medrano, 2002a; Tezara et al., 2002; Bota et al., 2004; Galmés et al., 2013). In the present study, the increased Rubisco activity accompanied by a decrease in the Rubisco content in WD-maize was associated with a higher Rubisco activation state, probably triggered by an increased ratio Rca/Rubisco (**Figure 5**).

Several authors have reported that Rubisco amount is highly affected by growth at HTs (Verlag et al., 2002; Gesch et al., 2003; Pérez et al., 2011). In the present study, the Rubisco amount was significantly lower at HT only for rice (**Figure 3**). However, large decreases in the Rubisco initial activity were observed at HT in all three species, which were not accompanied by changes in the Rubisco total activity. Overall, these data indicate that growth at HT induced a decrease in the Rubisco activation state in the three species. Further, the decrease in the Rubisco activation state caused a decrease in the photosynthetic capacity of the three crop species (**Figure 2**), in agreement with previous reports (Crafts-Brandner and Salvucci, 2000; Salvucci and Crafts-Brandner, 2004; Yamori and von Caemmerer, 2009; Scafaro et al., 2012). This decrease in the Rubisco activation state at HT was unrelated to variations in the total amount of Rubisco and Rca in any of the three species (**Figure 5**). Rubisco activity was measured at 25°C for both control and HT plants and some of the effects of mild-to-moderate heat stress on Rubisco activity and carbamylation state could have been lost when performing the assays at an optimal temperature (Galmés et al., 2013). However, others have also shown that temperature response of Rubisco activation does not appear to be strongly dependent on Rca content (Salvucci et al., 2006; Yamori and von Caemmerer, 2009). The total Rca amount remained unchanged across treatments in the three species (**Figure 4**), with the exception of wheat, for which Rca amount increased in the combined treatment HT-WD.

Rca is composed of small and large isoforms (Salvucci et al., 1987). Changes in the amount of the large Rca isoform in rice (slight increase) and wheat (slight decrease) at HT did not explain the large decreases in the Rubisco activation state (**Figures 4**, **5**). These results are consistent with the hypothesis that the intrinsic heat sensitivity of Rca is linked with the observed decrease in Rubisco activation (Salvucci and Crafts-Brandner, 2004; Barta et al., 2010; Carmo-Silva and Salvucci, 2011; Scafaro et al., 2016). On the other hand, decreased Rubisco activation state at HT correlated with the electron transport rate (J) in rice and wheat, irrespective of the watering treatment (**Figure 7**). This correlation did not hold for maize, a species that does not contain significant amounts of the large Rca isoform (Supplementary Figure S2; Salvucci et al., 1987). Lower J at HT may result in decreased ATP/ADP ratios and redox potential in the chloroplast, which in turn, could affect the activity of Rca and, consequently, the capacity to restore the activity of Rubisco (Zhang and Portis, 1999; Zhang et al., 2002; Sage and Kubien, 2007; Carmo-Silva et al., 2015). In addition to decreased J in plants grown at HT, Rca activity may be also affected by other processes which have not been measured in the present study and cannot be ruled out. In particular, at HTs protons can leak through the thylakoid membrane, impairing the coupling of ATP synthesis to electron transport (Bukhov et al., 1999, 2000; Pastenesz and Horton, 2014; Singh et al., 2014).

## Conclusion

In summary, photosynthesis was mainly affected by diffusive limitations under WD and by biochemical limitations at HT in rice, wheat and maize. Biochemical limitations were predominant also under the combination WD-HT in rice and maize. Increased biochemical limitations under HT were mainly attributed to decreased Rubisco activation state. In turn, decreased Rubisco activation was not related to altered amounts of Rca, but correlated with changes in the rate of electron transport. This result suggests that inhibited Rca activity was linked with the observed decrease in the Rubisco activation state, and ultimately, in the photosynthetic CO_2_ assimilation. Further research is required to verify whether increasing the thermal tolerance of Rca activity has the potential to increase photosynthesis at elevated temperatures. Since Rubisco activity impacts directly on the photosynthetic potential of plants, understanding the regulation of Rubisco and photosynthesis under heat stress is of pivotal importance to predict and mitigate consequences of future predicted climates on agriculture and natural ecosystems.

## Author Contributions

JAP performed the experiment, analyzed the data, and wrote the paper. SC-B contributed to the acquisition of the data. EC-S contributed to the design of the work, analysis and interpretation of the Rubisco activase data and to the preparation of the manuscript. JG obtained funding for the project, was a substantial contributor to the conception and design of the work and to the preparation of the manuscript.

## Conflict of Interest Statement

The authors declare that the research was conducted in the absence of any commercial or financial relationships that could be construed as a potential conflict of interest.
