# Supplementary material for: Rubisco and Rubisco Activase Play an Important Role in the Biochemical Limitations of Photosynthesis in Rice, Wheat, and Maize under High Temperature and Water Deficit
Source: Front Plant Sci. 2017 Apr 13;8:490. doi: 10.3389/fpls.2017.00490 (PMC5390490; doi:10.3389/fpls.2017.00490)
Supplement: Supplementary file 1 [file Table_1.DOCX]

***Supplementary Material***

**Rubisco and Rubisco activase play an important role in the biochemical limitations of photosynthesis in rice, wheat and maize under high temperature and water deficit.**

**Authors:** Juan Alejandro Perdomo^1^*, Elizabete Carmo-Silva^2^, Jeroni Galmés^3^

^1^Plant Biology and Crop Science, Rothamsted Research, Harpenden, AL5 2JQ, UK.

^2^Lancaster Environment Centre, Lancaster University, Lancaster, LA1 4YQ, UK.

^3^Research Group on Plant Biology under Mediterranean Conditions, Universitat de les Illes Balears, Balearic Islands, Spain.

***Corresponding author:** Juan Alejandro Perdomo

E-mail: alejandro.perdomo@rothamsted.ac.uk

Phone: +44 1582 938563.

**Table S1.** The gross photosynthesis (A_G_), the stomatal conductance (g_s_) and the mesophyll conductance (g_m_) measured at 25ºC in plants grown at 25ºC and well-watered conditions (control), 25ºC and water-deficit conditions (WD), 38ºC and well-watered conditions (HT) and 38ºC and water-deficit conditions (HT-WD). Values are means ± SE (n=4–5). This data was used, together with the *in vitro* Rubisco activity, in the analyses of photosynthetic limitations. For maize, g_m_ was considered constant across treatments at 2000 mmol m^-2^ s^-1^ (von Caemmerer, 2000).

| **Species** | **Treatments** | **A_G_**  **(µmol CO_2_ m^-2^ s^-1^)** | **g_s_**  **(mmol CO_2_ m^-2^ s^-1^)** | **g_m_**  **(mmol CO_2_ m^-2^ s^-1^)** |
| --- | --- | --- | --- | --- |
| Rice | Control | 26.6±2.1^c^ | 299±32^b^ | 439±86^b^ |
| Rice | WD | 11.6±1.5^a^ | 57±8^a^ | 187±46^a^ |
| Rice | HT | 19.0±0.6^b^ | 226±31^b^ | 197±13^a^ |
| Rice | HT-WD | 11.4±1.3^a^ | 125±21^a^ | 107±7^a^ |
| Wheat | Control | 24.2±0.9^b^ | 325±19^b^ | 198±4^a^ |
| Wheat | WD | 15.4±0.7^a^ | 59±5^a^ | 664±60^b^ |
| Wheat | HT | 21.5±1.0^b^ | 388±34^b^ | 143±9^a^ |
| Wheat | HT-WD | 14.4±1.0^a^ | 125±21^a^ | 106±8^a^ |
| Maize | Control | 27.3±1.9^c^ | 103±9^b^ | 2000 |
| Maize | WD | 19.4±0.4^b^ | 62±2^a^ | 2000 |
| Maize | HT | 12.1±1.4^a^ | 73±14^ab^ | 2000 |
| Maize | HT-WD | 11.2±3.4^a^ | 41±11^a^ | 2000 |

**Figure S1.** Acrylamide gels used to quantify Rubisco in rice, wheat and maize plants grown at 25ºC and well-watered conditions (ctrl), 25ºC and water-deficit conditions (WD), 38ºC and well-watered conditions (HT) and 38ºC and water-deficit conditions (HT-WD).


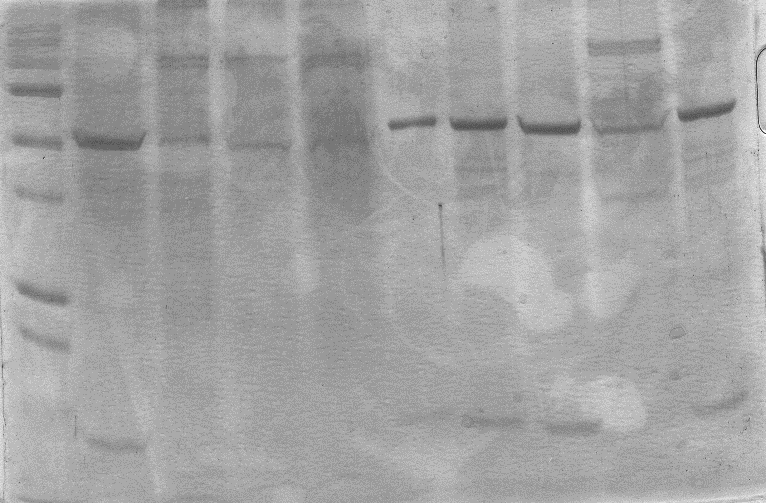

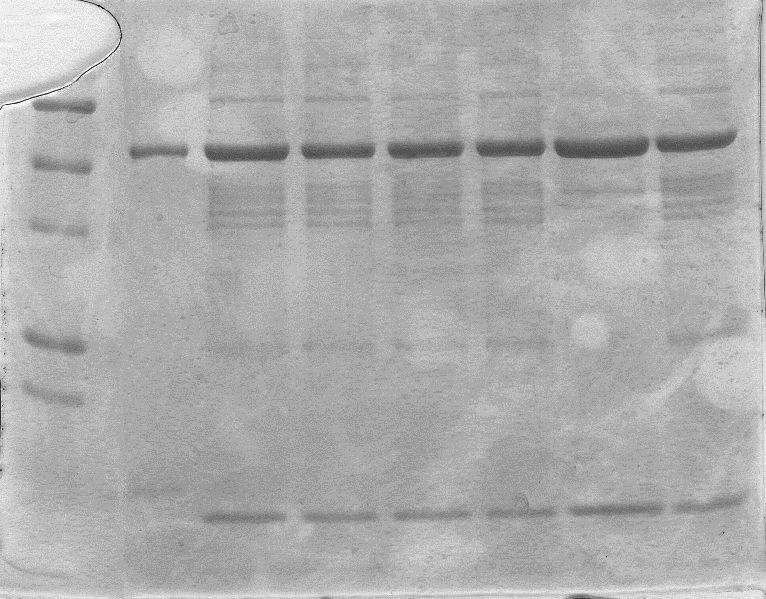


Wheat Ctrl

Maize Ctrl

Rice Ctrl


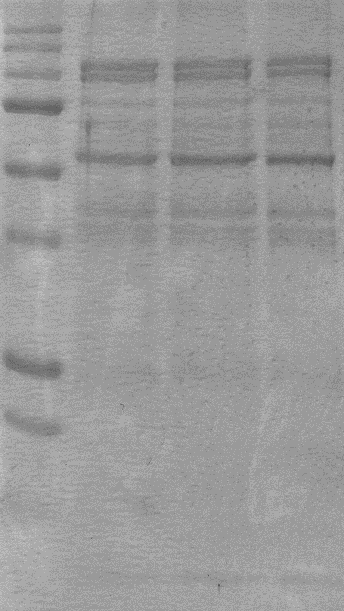

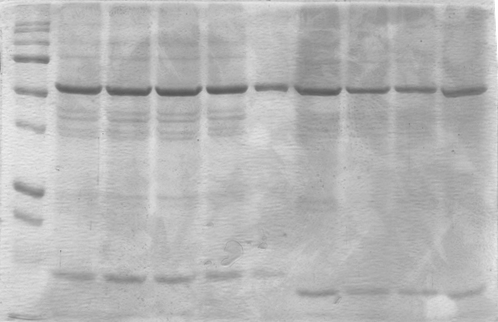


Rice WD

Maize WD

Wheat WD


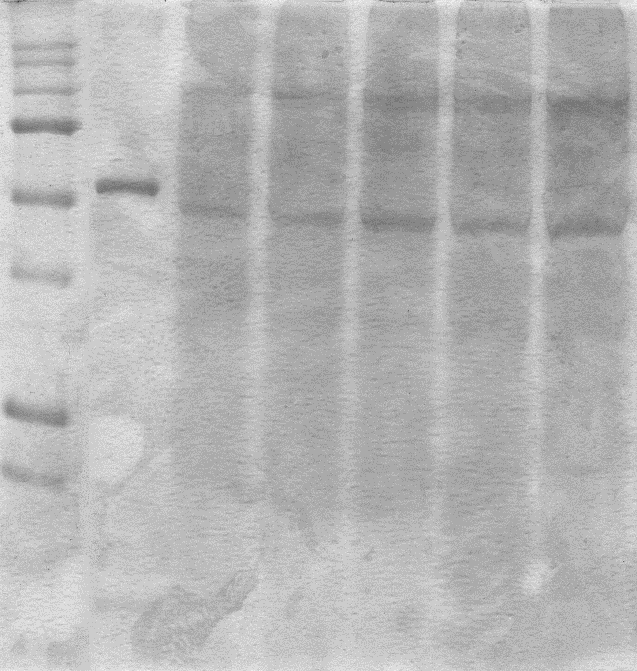

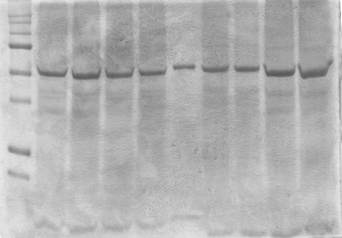


Wheat HT

Rice HT

Maize HT


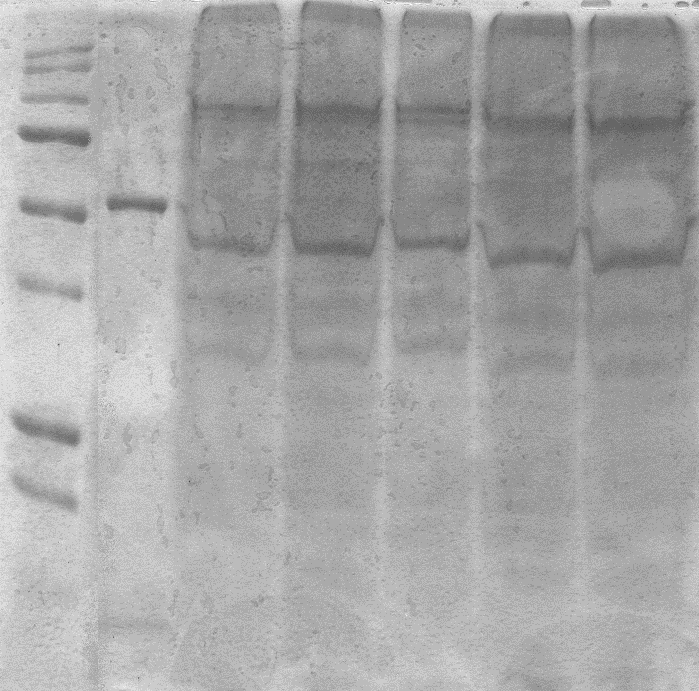




Wheat HT-WD

Maize HT-WD

Rice HT-WD

**Figure S2.** Western-blot membranes used to quantify Rubisco activase (Rca) in rice (A,B), wheat (C,D) and maize (E,F) plants grown at 25ºC and well-watered conditions (ctrl), 25ºC and water-deficit conditions (WD), 38ºC and well-watered conditions (HT) and 38ºC and water-deficit conditions (HT-WD). The standards (Std.) are a dilutions series of extracts prepared from leaf discs taken from plants of each species under control conditions and loading 5, 10 and 15 µg of TSP per well, respectively.


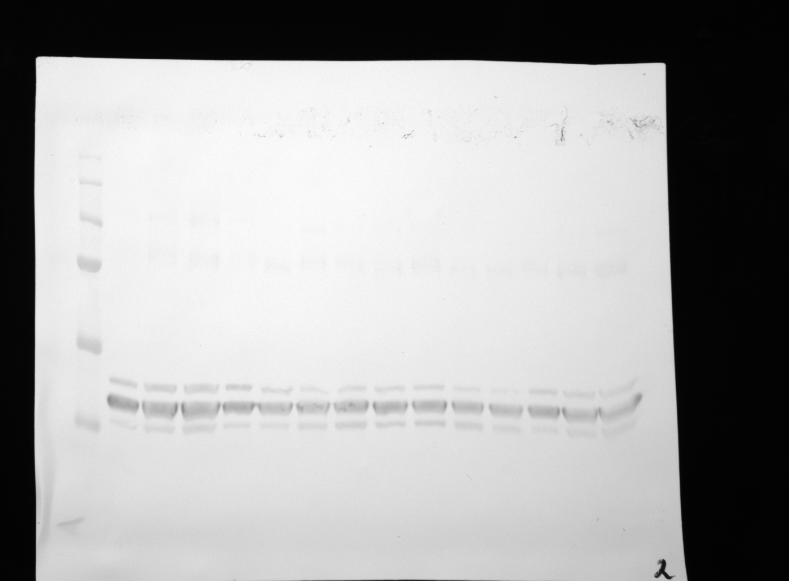

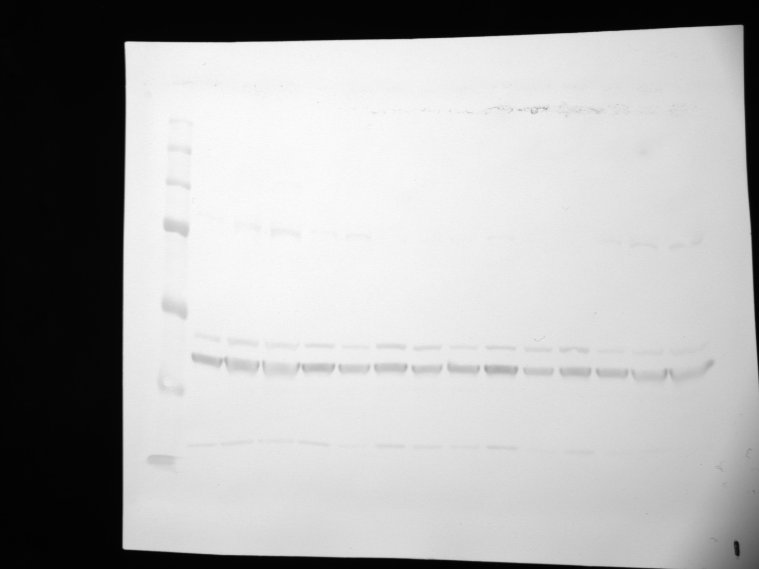
 A B

Std.

Std.

WD

HT-WD

HT-WD

Std.

Std.

WD

HT

Ctrl

HT

Ctrl


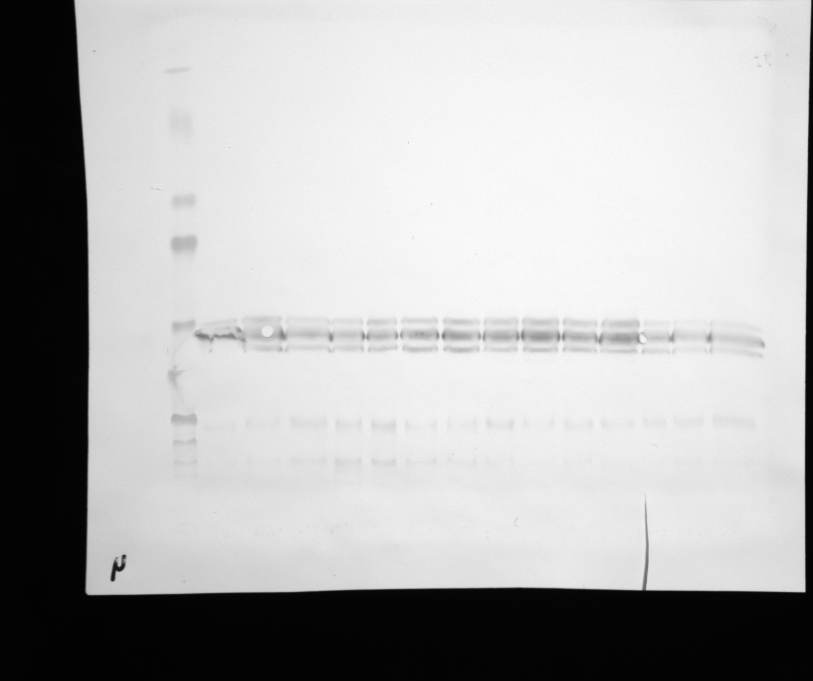

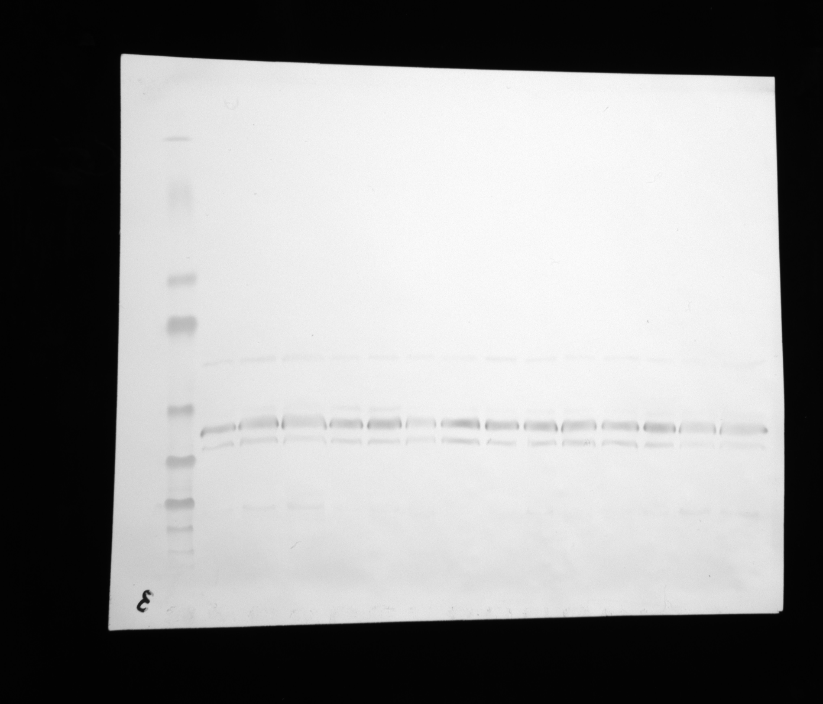


C D

Std.

HT

HT

Std.

Ctrl.

Ctrl.

HT-WD

HT-WD

WD

WD

Std.

Std.


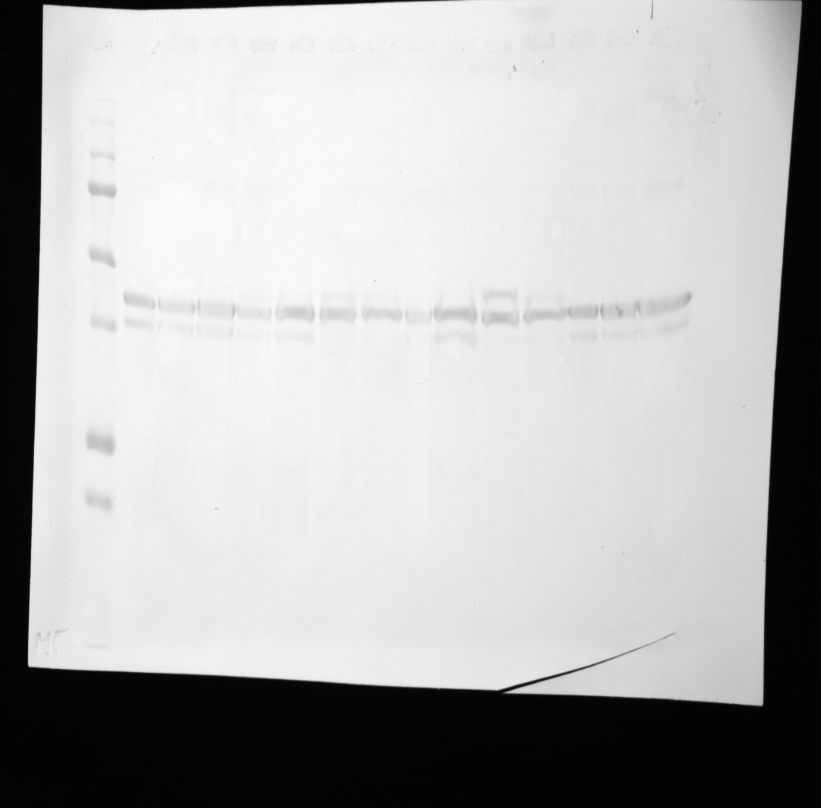

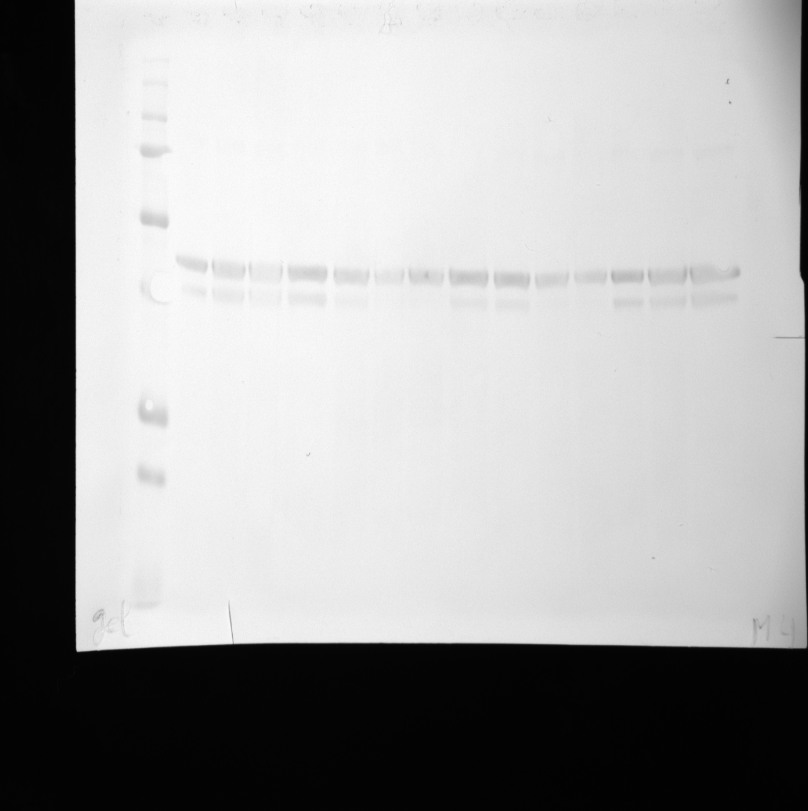
 E F

WD

WD

HT-WD

Std.

HT-WD

Std.

Std.

Ctrl

HT

Ctrl.

Std.

HT
